# L-Glutamine is better for treatment than prevention in exhaustive exercise

**DOI:** 10.3389/fphys.2023.1172342

**Published:** 2023-04-28

**Authors:** Cheng-Chung Lu, Chun-Yen Ke, Wen-Tien Wu, Ru-Ping Lee

**Affiliations:** ^1^ Institute of Medical Sciences, Tzu Chi University, Hualien, Taiwan; ^2^ Office of Physical Education, Hsing Wu University, New Taipei City, Taiwan; ^3^ Center for General Education, Cheng Shiu University, Kaohsiung, Taiwan; ^4^ Department of Orthopedics, Hualien Tzu Chi Hospital, Buddhist Tzu Chi Medical Foundation, Hualien, Taiwan; ^5^ School of Medicine, Tzu Chi University, Hualien, Taiwan

**Keywords:** exercise, muscle damage, glutamine, creatine kinase, treadmill

## Abstract

**Introduction:** Glutamine is known as the richest nonessential amino acid in the human body. The intake of glutamine is not only beneficial to nutrition but also reported to enhance inflammation reducing bioactivity in exercise. Although studies have demonstrated that glutamine is beneficial for exercise, the optimal intake timing remains unclear. This study examined whether the effects of glutamine on tissue damage and physiology differ between intake timings.

**Methods:** Rats were divided into without L-glutamine supplementation (vehicle), with L-glutamine before exhaustive exercise (prevention), and with L-glutamine after exhaustive exercise (treatment) groups. Exhaustive exercise was induced by treadmill running and L-glutamine was given by oral feeding. The exhaustive exercise began at a speed of 10 miles/min and increased in increments of 1 mile/min, to a maximum running speed of 15 miles/min with no incline. The blood samples were collected before exhaustive exercise, 12 h and 24 h after exercise to compare the creatine kinase isozyme MM (CK-MM), red blood cell count and platelet count. The animals were euthanized on 24 h after exercise, and tissue samples were collected for pathological examination and scored the severity of organ injury from 0 to 4.

**Results:** The CK-MM was elevated gradually after exercise in the vehicle group; however, CK-MM was decreased after L-glutamine supplementation in the treatment group. The treatment group had higher red blood cell count and platelet count than the vehicle and prevention group after exercise. In addition, the treatment group had less tissue injury in the cardiac muscles, and kidneys than prevention group.

**Conclusion:** The therapeutic effect of L-glutamine after exhaustive exercise was more effective than preventive before exercise.

## Introduction

Acute muscle damage often occurs after exhaustive exercise and also causes levels of lactatic acid, creatine kinase (CK), aspartate aminotransferase (AST), alanine aminotransferase (ALT), and lactate dehydrogenase in serum to increase (Baumert et al., 2016; Dupuy et al., 2018; Ra et al., 2018; Tokinoya et al., 2020). Exercise, especially exhaustive exercise, defined as exercise till unable to move, causes an inflammatory response and damage to multiple organs, including skeletal muscles, the respiratory system, the liver, and the kidneys (Ke et al., 2016). During exhaustive exercise, inflammatory responses such as leukocyte aggregation can be identified in muscle tissue (Peake et al., 2017). Muscle damage can occur when weaker sarcomeres are destroyed during muscle contraction or when a coupling mechanism stimulated through muscle excitation leads to excessive muscle lengthening (Negro et al., 2008; Peake et al., 2017). In addition, protein catabolism is greater than protein anabolism during exhaustive exercise. In the metabolic pathway of protein catabolism, the nitrogen-containing pathway must first undergo transamination (Gleeson, 2008; Coqueiro et al., 2019a). Glutamine in the muscle is converted into glutamic acid to facilitate transamination, and the glutamine concentration in the body thus decreases after exhaustive exercise (Trivedi et al., 2022). Therefore, a moderate amount of glutamine supplementation may assist in reducing muscle damage resulting from exhaustive exercise (Gleeson, 2008).

Glutamine is not only nutritious but also able to regulate other bioactivities in the body. The animal study indicates that oral supplementation with L-glutamine and alanine in the free form can effectively maintain glutamine stores, leading to lower levels of proinflammatory biomarkers such as TNF-a, IL-1β and IL-6 (Cruzat et al., 2010). Additionally, the study shows that these supplementations can also have beneficial effects on biomarkers of muscle damage and inflammation such as muscle glycogen and plasma creatine kinase isozyme (CK) after a period of training. The study also found that while glutamine and alanine supplementation improved some fatigue markers, it did not improve exercise performance (Coqueiro et al., 2019a). Moreover, glutamine affects many bioactivities in the digestive system and the immune system in the human body. In the digestive system, glutamine supplements can accelerate rapidly dividing cells and reduce splanchnic bed damage in the intestines, liver, and pancreas (Coqueiro et al., 2019b). In the immune system, glutamine downregulates lymphocyte count, macrophage count, and the expression of proinflammatory cytokines, such as interlukin-1 (IL-1), IL-2, Interferon-gamma (Amin et al., 2021; Zhang et al., 2021). Glutamine supplementation for human exercise was demonstrated to effectively reduce tissue and organ damage and promote glycogen synthesis, providing nutritional support for the immune system, and preventing infection (Bowtell et al., 1999; Negro et al., 2008; American Dietetic Association et al., 2009). Muscle metabolism parameters such as creatine kinase isozyme MM (CK-MM) were used as an indicator for muscle damage. The serum level of CK-MM typically increases after exercise (Banfi et al., 2012; Koch et al., 2014). During exercise, local hypoxia in skeletal muscles, accumulation of metabolites, and an increase in free radicals cause cell membrane damage and increased permeability. At this time, CK-MM in muscle cells penetrates the cell membrane and enters the blood, which is the same process that leads to an increase of CK-MM after exhaustive exercise (Koch et al., 2014; Moghadam-Kia et al., 2016). Some additional biomarkers affected by exercise are red blood cell count (RBC) and hematocrit (HCT); after exhaustive exercise, hemolysis and decreased HCT are observed, a condition commonly called “sports anemia” (Mairbäurl, 2013; Lippi and Sanchis-Gomar, 2019). Platelet count also decreases after exercise and this decrease might be related to exercise-based cardiac rehabilitation that decreases mortality in patients with coronary artery disease (Anz et al., 2019; Durmuş et al., 2021).

Researchers also have reported that supplementation after exercise exerts many beneficial effects; especially in muscle recovery and connective tissue damage (Grassi et al., 2018; Anz et al., 2019; Scott et al., 2019; Vander Doelen and Jelley, 2020). Although studies have demonstrated that glutamine has beneficial effects for exercise, a crucial factor remains unclear. Researchers have been focused on the effect of glutamine on immunoregulation and muscle damage during exercise, and no other organ pathology observation for the use of glutamine has been undertaken. Furthermore, no study has discussed whether various intake timings affect glutamine bioactivity in exhaustive exercise. If glutamine supplementation has a treatment effect after exercise, then glutamine supplementation may be has a preventive effect before exercise. Nevertheless, there is no research show which is better to have glutamine supplementation before or after exercise. Thus, the purpose of this study was to examine the differential effect of glutamine supplementation before and after exercise to identify the optimal timing of glutamine supplementation. We hypothesize that glutamine supplementation before exercise may have a similar effect to after exercise. Besides, glutamine regulates not only the immune cells but also other tissues or blood cells, such as RBC and platelets.

## Methods

In this study, animal experimentation was conducted to explore the difference in the effect of L-glutamine supplementation at different times (i.e., before and after exercise). The Sprague–Dawley rat model was applied. The eighteen experimental animals were randomly divided into three groups, namely, the vehicle group (no glutamine treatment), the prevention group (administered glutamine before exhaustive exercise), and the treatment group (administered glutamine after exercise). The three groups engaged in the same exercise program. Blood samples were collected at different times, and CK-MM was tested to assess muscle tissue damage. After the experiment, heart, kidney, and liver tissue sections were dissected to measure tissue damage. The animal study was performed according to institutional protocols of the Institutional Animal Care and Use Committee of Tzu Chi University (IACUC No: 103085).

### Experiment protocol

Experimental rats, with body weights between 260 and 280 g, were ordered from LASCO animal center (Taipei, Taiwan). The rats were housed in a controlled environment of 22°C ± 1°C with a 12 h light-dark cycle. Food pellets and water were provided *ad libitum*. Before the beginning of the 14-day experiment, the rats were trained to exercise on a treadmill. During this period, the rats exercised 20 min/day at a speed of 10 miles/min with no incline. After 14 days, the rats were transitioned to exercising on a treadmill and underwent exhaustive exercise. The rats were randomly grouped into a vehicle group, treatment group, and prevention group. The vehicle group received 0.5 mL distilled water feeding 1 hour after the exercise. The treatment group received a single-dose 1 g/kg glutamine dissolved in distilled water feeding 1 hour after the exercise. The actual mean dose of L-glutamine administered was between 0.26 mg to 0.28 mg. The prevention group received the same single-dose glutamine 1 hour before the exercise. The rats were anesthetized using 2% isoflurane inhalation (Baxter Healthcare of Puerto Rico, Guayama, Puerto Rico) during a 15 min surgical procedure 1 h after the exhaustive exercise was performed. During this period, polyethylene catheters (PE-50) were inserted into the right femoral artery to collect blood samples (Ke et al., 2016). The femoral artery catheter was also connected to an electrophysiological amplifier (Gould Instruments, Cleveland, OH, United States) to monitor arterial pressure and heart rate. The surgical incision was less than 0.5 cm^2^, and all surgical procedures were performed under sterile conditions. After the surgery, the animals were placed in a metabolic cage and awakened soon thereafter. The rats were allowed free access to food and water.

### Exercise procedure

The exhaustive exercise began at a speed of 10 miles/min and increased in increments of 1 mile/min, to a maximum running speed of 15 miles/min with no incline. Maximum running times were attained for each rat, and the maximum running time was 30 min. Exhaustion was defined in accordance per previous studies (Ke et al., 2016). Specifically, exhaustion was concluded to have occurred when the rat was unable to maintain pace with the treadmill and when the rat lay flat on the treadmill and remained on the grid at the back of the treadmill for a period of 30 s despite being gently pushed with sticks or breathed upon.

### Blood cell counts and level of skeletal muscle-specific creatine kinase in blood

The blood samples were collected before exercise, 12 h and 24 h after exercise. These samples were placed into heparinized tubes and measured immediately for blood cell counts (Sysmex K-1000, NY, United States). The samples were then centrifuged at 3,000 × g for 10 min. After centrifugation, supernatant was collected and the level of CK-MM was measured within 1 hour by using an automatic biochemical analyzer (COBAS INTEGRA 800, Roche Diagnostics, Basel, Switzerland).

### Histological examination

Euthanasia was conducted 24 h after treatments. The rats were deeply anesthetized using isoflurane inhalation and then blood withdrawal was performed for euthanasia. The heart, kidneys, and liver were removed immediately. These tissue specimens were submerged overnight in 4% buffered formaldehyde, processed using standard methods, and stained with HE stains. An observer blinded to the group allocations performed the tissue analysis and scored the severity of organ injury. The severity of heart injuries observed in the tissue sections was scored from 0 (minimal or no evidence of injury) to 4 (>75% damaged). The severity of renal tubular injuries was scored by estimating the percentage of tubules in the cortex or the outer medulla that exhibited epithelial necrosis or luminal necrotic debris, tubular dilation, and hemorrhage, as follows: 0, none; 1, <5%; 2, 5%–25%; 3, 25%–75%; and 4, >75% (Ke et al., 2016). The severity of liver injuries observed in the tissue sections was scored as follows: 0, minimal or no evidence of injury; 1, mild injury consisting of cytoplasmic vacuolation and focal nuclear pyknosis; 2, moderate to severe injury with extensive nuclear pyknosis, cytoplasmic hypereosinophilia, and loss of intercellular borders; and 3, severe necrosis with disintegration of the hepatic cords, hemorrhage, and neutrophil infiltration (Ke et al., 2016). All evaluations were performed on five fields per section and five sections per organ by a blinding observer.

### Statistical analysis

The collected data were analyzed using SPSS for Windows v22.0 (IBM, NJ, United States). The continuous variables have been expressed in Mean ± SD, and an independent *t*-test was employed to analyze and compare the tissue injury score between prevention and treatment group. The analysis of variance (ANOVA) was applied to examine the differences of CK-MM and blood cell count between three groups. The significance level for all statistical comparisons was set as α less than or equal to 0.05 (2-tail).

## Results

### Differences of CK-MM level

**FIGURE 1:**
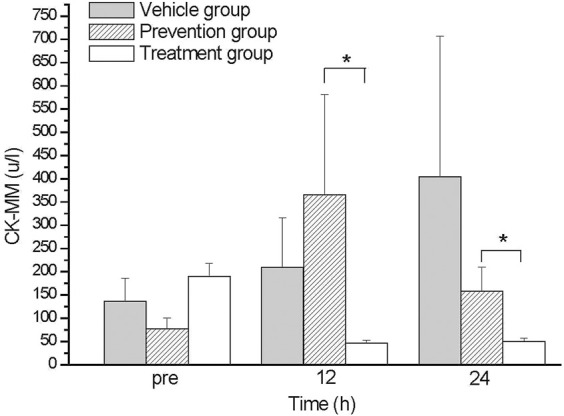
The comparison of CK-MM levels between groups. The CK-MM levels of treatment group were lower than those in prevention group at 12 and 24 hs. *indicates *p*-value <0.05, compare with prevention group.

The skeletal muscle damage biomarker CK-MM was analyzed. The CK-MM level of the vehicle group was elevated during the time course from 136 u/L at baseline (pre) to 404 u/L at 24 h (p24) after exercise (Figure 1). The prevention group exhibited a higher serum CK-MM level at 12 h (p12); the average value during this duration was 365 u/L and then decreased to 151 u/L at 24 h. The treatment group had a lower serum CK-MM level compared with other groups, which had a level 48 u/L at 24 h (Figure 1). The data showed that timing of the oral intake affects the reduction of serum CK-MM level. The CK-MM level of untreated vehicle group was elevated after exhaustive exercise and reached its highest point at 24 h after exercise (Figure 1). The serum CK-MM level of the prevention group was elevated at 12 h. The treatment group exhibited almost no elevation.

### Differences of blood count analysis

**FIGURE 2:**
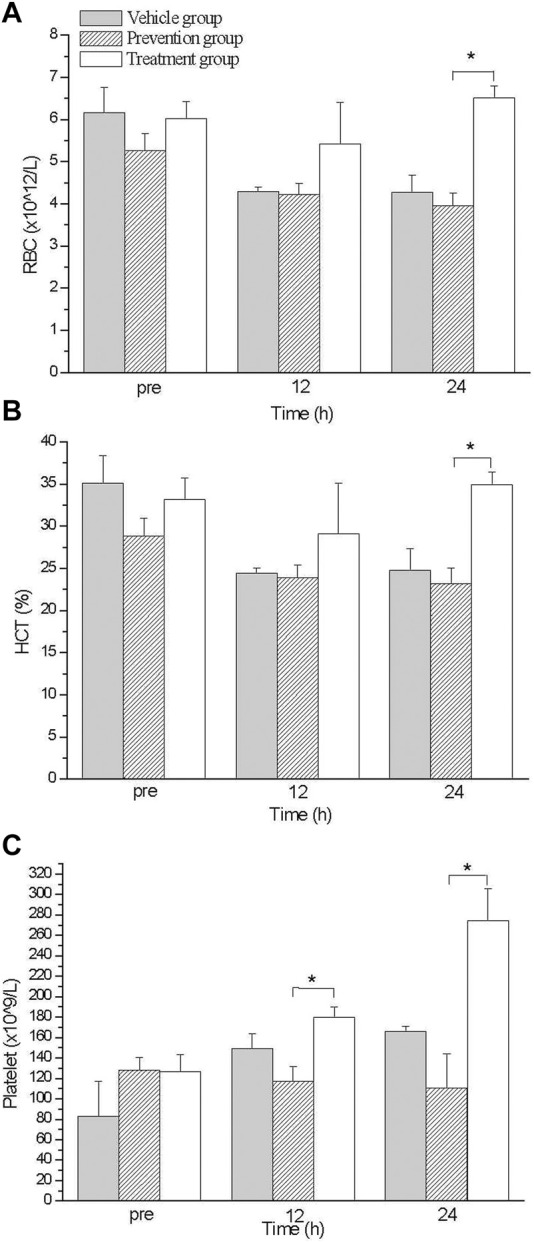
The comparison of blood count levels between groups. Treatment group had higher RBC **(A)**, HCT **(B)** and PLT **(C)** values than those in prevention group at 24 hs. *indicates *p*-value <0.05, compare with prevention group.

Complete blood count analysis was performed at various time points after exercise treatment. Circulation RBC was declining after exercise treatment in all three group, which continued up to 12 h after exercise; however, after 12 h, the treatment group’s RBC level was substantially elevated compared with other groups, from 5.4 012/L to 6.5 1,012/L; the RBC levels of the other groups were maintaining at 4.0 (prevention group) to 4.3 (vehicle group) 1,012/L (Figure 2A). Hematocrit showed a similar trend to RBC. The average HCT was significantly higher in the treatment group 12 h after exercise and reached a maximum difference at 24 h. The average HCT of the treatment group in p24 was 35%, and those in the other two groups were 24.8% (vehicle group) and 23.1% (prevention group) (Figure 2B). We also observed that PLT improved to a similar extent as RBC did. The average PLT was considerably higher in the treatment group after exercise 12 h and had a maximum improvement at 24 h. The average PLT of the treatment group in p24 was 274.7 × 109/L, and those in the other two groups were 165.8 × 109/L (vehicle group) and 110.7 × 109/L (prevention group) (Figure 2C). In this part, we found that oral intake of glutamine elevated RBC, HCT, and PLT amounts only in the treatment group.

### The histological examination

**FIGURE 3:**
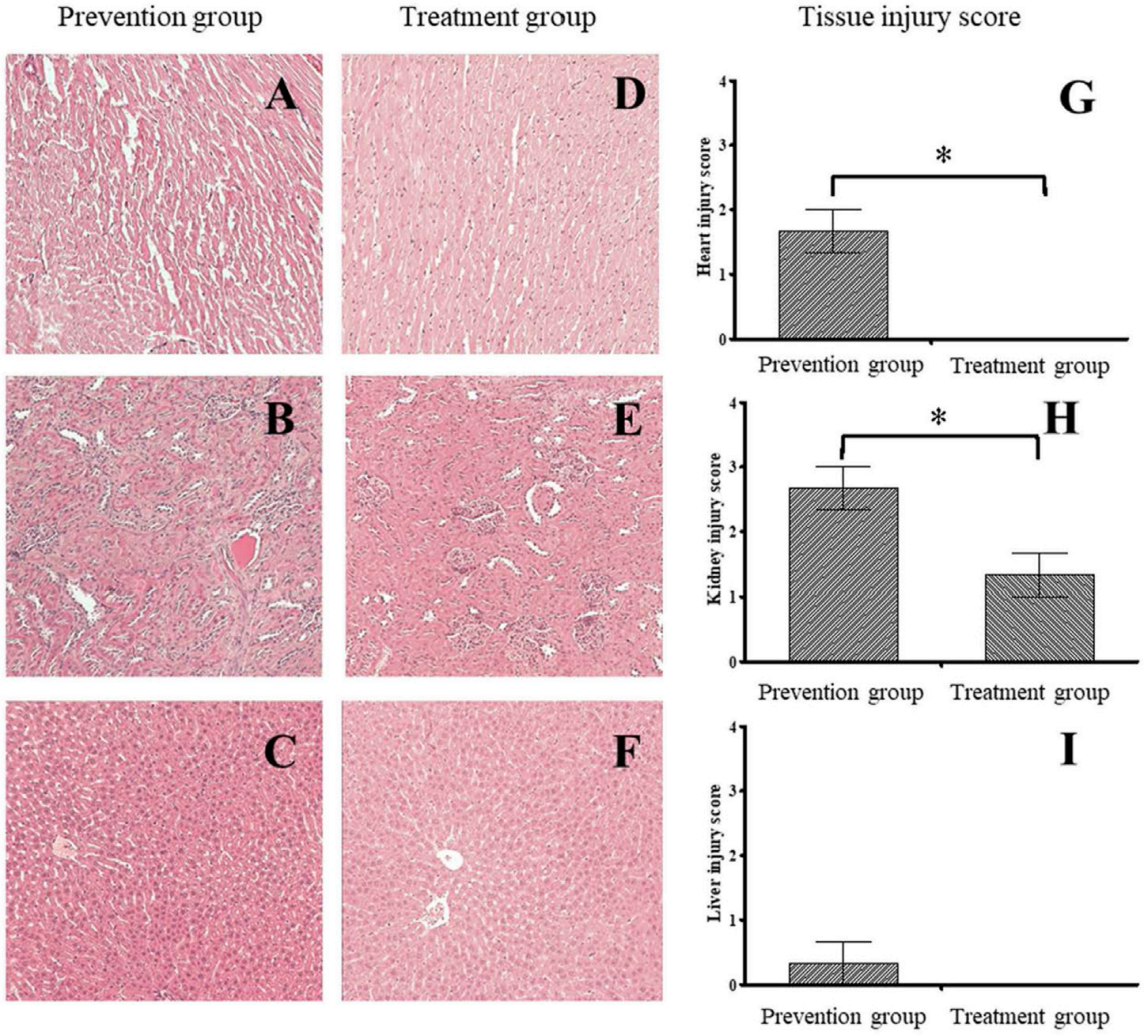
The histological finding and tissue injury score between prevention and treatment group. The histological examination of prevention **(A–C)** and treatment group **(D–F)**. The prevention group had higher tissue injury score on the Heart **(G)** and kidney **(H)** than those in treatment group. There was no significant difference between groups on the liver **(I)**. *indicates *p*-value <0.05, compare with prevention group.

The HE staining results indicated tissue injuries in the cardiac muscles (Figure 3A), kidneys (Figure 3B), and liver (Figure 3C) in the prevention group, with average respective scores of 1.67 (Figure 3G), 2.67 (Figure 3H), and 0.33 (Figure 3I). The treatment group had less tissue injury in the cardiac muscles (Figure 3D), kidneys (Figure 3E), and liver (Figure 3F), with average respective scores of 0 (Figure 3G), 1.33 (Figure 3H), and 0 (Figure 3I). These data of histological examination indicated that the treatment group had a more substantial reduction in damage, especially cardiac and renal damage, compared with the prevention group (Figure 3).

## Discussion

We found that glutamine can reduce skeletal muscle damage caused by exhaustive exercise, and the treatment group had a greater reduction in damage than the prevention group did (Figure 1). We also found that oral intake of glutamine elevated RBC, HCT, and PLT amounts only in the treatment group (Figure 2). A histological examination indicated that the treatment group had a more substantial reduction in damage, especially cardiac and renal damage, compared with the prevention group (Figure 3). The results of this study indicated that the effect of supplementing L-glutamine after exercise was more satisfactory than that before exercise. The tissue section results demonstrated that glutamine not only protected muscles under exhaustive exercise but also prevented damage to specific organs.

After exhaustive exercise, because of the continued contraction of skeleton muscles and enhanced circulation stress, both skeletal and cardiac muscles are damaged. Markers of skeletal and cardiac muscle damage, such as serum biomarker CK-MM, indicated damage after exercise, and the damage could also be found in histopathology examinations (Amelink et al., 1991; Cruzat et al., 2010; Beck et al., 2015). Studies have demonstrated that supplementation with glutamine has beneficial effects on reducing the parameters of muscle damage and inflammation in exercise rats (Bowtell et al., 1999; Gleeson, 2008; Cruzat et al., 2010). Similarly, in our study, the serum CK-MM level of the untreated vehicle group was elevated after exhaustive exercise and reached its highest point at 24 h after exercise (Figure 1). The serum CK-MM level of the prevention group was elevated at 12 h. The treatment group exhibited almost no elevation. Decreased glutamine concentrations typically correlate with the severity of the underlying disease process, with large amounts of glutamine catabolized in muscle at the time of damage. Concentrations only gradually recover in the later stage of healing (Durkalec-Michalski et al., 2021). During exhaustive exercise, the protein metabolism of muscles increases. At this time, glutamine can assist in gluconeogenesis, generating glucose to be used by the muscles, promoting energy metabolism and antioxidant capacity, reducing organ damage, and contributing to the synthesis and repair of muscle tissue. Under exhaustive exercise, glutamine in the body is used in substantial quantities, resulting in a decreased glutamine concentrations (Afonso et al., 2021; Sonkodi et al., 2021). We further found that the intake timing will notably affect the beneficial effect of glutamine for exhaustive exercise. This might cause by a short half-life of glutamine. Glutamine was more effective when taken orally after rather than before exhaustive exercise.

We also found that the RBC level increased substantially upon the post-exercise intake of glutamine (Figure 2). Previous researchers have demonstrated that glutamine is an essential source for glutathione synthesis in human erythrocytes (Whillier et al., 2011). During exhaustive exercise, because of elevated oxidative stress and overloaded cardiac output, RBC becomes oxidative damaged (Smith, 1995). No study has discussed whether glutamine mitigates oxidative damage on RBC or enhances the regeneration of RBC after exhaustive exercise. We found that the RBC level increase and accordingly glutamine might enhance the regeneration of RBC upon oral intake after exercise (Figure 2). The differences between the prevention and treatment groups were not only with respect to RBC concentration, but also regarding tissue damage for histological examinations. The treatment group showed more considerable damage reduction in the cardiac muscle and kidneys than the prevention group showed. (Figures 3G, H) The primary cause for this difference in damage reduction might be the maldigestion of glutamine during exercise. Eating before exhaustive exercise often causes maldigestion; furthermore, body temperature increases during exercise until rest. Research has demonstrated that in a hot environment, intestinal permeability is reduced by glutamine supplementation (Pugh et al., 2017). Therefore, glutamine intake is more beneficial after rather than before exhaustive exercise. Relative to the prevention group, the treatment group had a more considerable reduction in the damage to their cardiac muscles and kidneys. Our results revealed that the timing of glutamine oral intake influences outcomes, such as improved organs protection and elevated RBC concentration in blood. Although the conditions of sports practice in humans are far different from those that can be applied in laboratory rats, these results might suggest athletes take supplements at the proper timing after exhaustive exercise.

## Conclusion

Daily supplementation of L-glutamine can reduce the skeletal muscle damage caused by exhaustive exercise and that the timing of the oral intake affects the reduction. Glutamine as a treatment more considerably reduced damage than it had as a prophylactic. We also observed that the oral intake of glutamine could elevate RBC, HCT, and PLT only after exhaustive exercise. It seems like the proper timing for taking glutamine supplements is after exercise. However, the further clinical trial is needed in the future study.

## Data Availability

The original contributions presented in the study are included in the article/supplementary material, further inquiries can be directed to the corresponding author.

## References

[B1] Afonso J. Clemente F. M. Nakamura F. Y. Morouço P. Sarmento H. Inman R. A. (2021). The effectiveness of post-exercise stretching in short-term and delayed recovery of strength, range of motion and delayed onset muscle soreness: A systematic review and meta-analysis of randomized controlled trials. Front. Physiol. 12, 677581. 10.3389/fphys.2021.677581 34025459 PMC8133317

[B2] Amelink G. J. van der Wal W. A. A. Wokke J. H. J. van Asbeck B. S. Bär P. R. (1991). Exercise-induced muscle damage in the rat: The effect of vitamin E deficiency. Pflügers. Arch. 419 (3), 304–309. 10.1007/BF00371111 1745605

[B26] American Dietetic Association Dietitians of Canada American College of Sports Medicine Rodriguez N. R. Di Marco N. M. Langley S. Langley S. (2009). American College of Sports Medicine position stand. Nutrition and athletic performance. Med. Sci. Sports Exerc. 41 (3), 709–731. 10.1249/MSS.0b013e31890eb86 19225360

[B3] Amin M. N. El-Mowafy M. Mobark A. Abass N. Elgaml A. (2021). Exercise-induced downregulation of serum interleukin-6 and tumor necrosis factor-alpha in Egyptian handball players. Saudi J. Biol. Sci. 28 (1), 724–730. 10.1016/j.sjbs.2020.10.065 33424360 PMC7783837

[B4] Anz A. W. Parsa R. S. Romero-Creel M. F. Nabors A. Tucker M. S. Harrison R. M. (2019). Exercise-mobilized platelet-rich plasma: Short-term exercise increases stem cell and platelet concentrations in platelet-rich plasma. Arthroscopy 35 (1), 192–200. 10.1016/j.arthro.2018.06.043 30611351

[B5] Banfi G. Colombini A. Lombardi G. Lubkowska A. (2012). Metabolic markers in sports medicine. Adv. Clin. Chem. 56, 1–54. 10.1016/b978-0-12-394317-0.00015-7 22397027

[B6] Baumert P. Lake M. J. Stewart C. E. Drust B. Erskine R. M. (2016). Genetic variation and exercise-induced muscle damage: Implications for athletic performance, injury and ageing. Eur. J. Appl. Physiol. 116 (9), 1595–1625. 10.1007/s00421-016-3411-1 27294501 PMC4983298

[B7] Beck W. R. Botezelli J. D. Pauli J. R. Ropelle E. R. Gobatto C. A. (2015). Melatonin has an ergogenic effect but does not prevent inflammation and damage in exhaustive exercise. Sci. Rep. 5, 18065. 10.1038/srep18065 26669455 PMC4680866

[B8] Bowtell J. L. Gelly K. Jackman M. L. Patel A. Simeoni M. Rennie M. J. (1999). Effect of oral glutamine on whole body carbohydrate storage during recovery from exhaustive exercise. J. Appl. Physiol. 86 (6), 1770–1777. 10.1152/jappl.1999.86.6.1770 10368336

[B9] Coqueiro A. Y. Raizel R. Bonvini A. Rogero M. M. Tirapegui J. (2019a). Effects of glutamine and alanine supplementation on muscle fatigue parameters of rats submitted to resistance training. Nutrition 65, 131–137. 10.1016/j.nut.2018.09.025 31100607

[B10] Coqueiro A. Y. Rogero M. M. Tirapegui J. (2019b). Glutamine as an anti-fatigue amino acid in sports nutrition. Nutrients 11 (4), 863. 10.3390/nu11040863 30999561 PMC6520936

[B11] Cruzat V. F. Rogero M. M. Tirapegui J. (2010). Effects of supplementation with free glutamine and the dipeptide alanyl-glutamine on parameters of muscle damage and inflammation in rats submitted to prolonged exercise. Cell biochem. Funct. 28 (1), 24–30. 10.1002/cbf.1611 19885855

[B12] Dupuy O. Douzi W. Theurot D. Bosquet L. Dugué B. (2018). An evidence-based approach for choosing post-exercise recovery techniques to reduce markers of muscle damage, soreness, fatigue, and inflammation: A systematic review with meta-analysis. Front. Physiol. 9, 403. 10.3389/fphys.2018.00403 29755363 PMC5932411

[B13] Durkalec-Michalski K. Kusy K. Główka N. Zieliński J. (2021). The effect of multi-ingredient intra-versus extra-cellular buffering supplementation combined with branched-chain amino acids and creatine on exercise-induced ammonia blood concentration and aerobic capacity in taekwondo athletes. J. Int. Soc. Sports Nutr. 18 (1), 48. 10.1186/s12970-021-00451-3 34127014 PMC8204562

[B14] Durmuş İ. Kalaycıoğlu E. Çetin M. Şahin H. B. Kırış T. (2021). Exercise-based cardiac rehabilitation has a strong relationship with mean platelet volume reduction. Arq. Bras. Cardiol. 116 (3), 434–440. 10.36660/abc.20190514 33566933 PMC8159561

[B15] Gleeson M. (2008). Dosing and efficacy of glutamine supplementation in human exercise and sport training. J. Nutr. 138 (10), 2045S–2049S. 10.1093/jn/138.10.2045S 18806122

[B16] Grassi A. Napoli F. Romandini I. Samuelsson K. Zaffagnini S. Candrian C. (2018). Is platelet-rich plasma (prp) effective in the treatment of acute muscle injuries? A systematic review and meta-analysis. Sports Med. 48 (4), 971–989. 10.1007/s40279-018-0860-1 29363053

[B17] Ke C. Y. Yang F. L. Wu W. T. Chung C. H. Lee R. P. Yang W. T. (2016). Vitamin D3 reduces tissue damage and oxidative stress caused by exhaustive exercise. Int. J. Med. Sci. 13 (2), 147–153. 10.7150/ijms.13746 26941574 PMC4764782

[B18] Koch A. J. Pereira R. Machado M. (2014). The creatine kinase response to resistance exercise. J. Musculoskelet. Neuronal Interact. 14 (1), 68–77. 24583542

[B19] Lippi G. Sanchis-Gomar F. (2019). Epidemiological, biological and clinical update on exercise-induced hemolysis. Ann. Transl. Med. 7 (12), 270. 10.21037/atm.2019.05.41 31355237 PMC6614330

[B20] Mairbäurl H. (2013). Red blood cells in sports: Effects of exercise and training on oxygen supply by red blood cells. Front. Physiol. 4, 332. 10.3389/fphys.2013.00332 24273518 PMC3824146

[B21] Moghadam-Kia S. Oddis C. V. Aggarwal R. (2016). Approach to asymptomatic creatine kinase elevation. Cleve. Clin. J. Med. 83 (1), 37–42. 10.3949/ccjm.83a.14120 26760521 PMC4871266

[B22] Negro M. Giardina S. Marzani B. Marzatico F. (2008). Branched-chain amino acid supplementation does not enhance athletic performance but affects muscle recovery and the immune system. J. Sports Med. Phys. Fit. 48 (3), 347–351. 18974721

[B23] Peake J. M. Neubauer O. Della Gatta P. A. Nosaka K. (2017). Muscle damage and inflammation during recovery from exercise. J. Appl. Physiol. 122 (3), 559–570. 10.1152/japplphysiol.00971.2016 28035017

[B24] Pugh J. N. Sage S. Hutson M. Doran D. A. Fleming S. C. Highton J. (2017). Glutamine supplementation reduces markers of intestinal permeability during running in the heat in a dose-dependent manner. Eur. J. Appl. Physiol. 117 (12), 2569–2577. 10.1007/s00421-017-3744-4 29058112 PMC5694515

[B25] Ra S. G. Miyazaki T. Kojima R. Komine S. Ishikura K. Kawanaka K. (2018). Effect of BCAA supplement timing on exercise-induced muscle soreness and damage: A pilot placebo-controlled double-blind study. J. Sports Med. Phys. Fit. 58 (11), 1582–1591. 10.23736/S0022-4707.17.07638-1 28944645

[B27] Scott A. LaPrade R. F. Harmon K. G. Filardo G. Kon E. Della Villa S. (2019). Platelet-rich plasma for patellar tendinopathy: A randomized controlled trial of leukocyte-rich prp or leukocyte-poor prp versus saline. Am. J. Sports Med. 47 (7), 1654–1661. 10.1177/0363546519837954 31038979

[B28] Smith J. A. (1995). Exercise, training and red blood cell turnover. Sports Med. 19 (1), 9–31. 10.2165/00007256-199519010-00002 7740249

[B29] Sonkodi B. Kopa Z. Nyirády P. (2021). Post orgasmic illness syndrome (POIS) and delayed onset muscle soreness (DOMS): Do they have anything in common? Cells 10 (8), 1867. 10.3390/cells10081867 34440637 PMC8392034

[B30] Tokinoya K. Ishikura K. Ra S. G. Ebina K. Miyakawa S. Ohmori H. (2020). Relationship between early-onset muscle soreness and indirect muscle damage markers and their dynamics after a full marathon. J. Exerc Sci. Fit. 18 (3), 115–121. 10.1016/j.jesf.2020.03.001 32351588 PMC7183207

[B31] Trivedi K. Hussain M. S. Mohapatra C. (2022). Role of Glutamine as an ergogenic amino acid during fatigue. J. Clin. Med. Rev. Rep. 4 (2), 1–6. 10.31579/2690-8794/111

[B32] Vander Doelen T. Jelley W. (2020). Non-surgical treatment of patellar tendinopathy: A systematic review of randomized controlled trials. J. Sci. Med. Sport. 23 (2), 118–124. 10.1016/j.jsams.2019.09.008 31606317

[B33] Whillier S. Garcia B. Chapman B. E. Kuchel P. W. Raftos J. E. (2011). Glutamine and α-ketoglutarate as glutamate sources for glutathione synthesis in human erythrocytes. FEBS J. 278 (17), 3152–3163. 10.1111/j.1742-4658.2011.08241.x 21749648

[B34] Zhang H. Chen T. Ren J. Xia Y. Onuma A. Wang Y. (2021). Pre-operative exercise therapy triggers anti-inflammatory trained immunity of Kupffer cells through metabolic reprogramming. Nat. Metab. 3 (6), 843–858. 10.1038/s42255-021-00402-x 34127858 PMC8462058

